# Triglycerides as a biological marker of repeated re-hospitalization resulting from deliberate self-harm in acute psychiatry patients: a prospective observational study

**DOI:** 10.1186/1471-244X-14-54

**Published:** 2014-02-25

**Authors:** John O Roaldset, Olav M Linaker, Stål Bjørkly

**Affiliations:** 1 Psychiatric Department, Ålesund Hospital, Møre & Romsdal Health Trust, Alesund 6026, Norway; 2 Institute of Neuromedicine, Faculty of Medicine, The Norwegian University of Science and Technology, Trondheim 7489, Norway; 3 Centre for Research and Education in Forensic Psychiatry, Oslo University Hospital, Nydalen, Box 4956, Oslo 0424, Norway; 4 Psychiatric Department, Trondheim University Hospital, Lade, Box 3008, Trondheim 7441, Norway; 5 Institute of Health and Social Sciences, Molde University College, Box 2110, Molde 6402, Norway

**Keywords:** Prospective, Suicidal behavior, Deliberate self-harm (DSH), Bio-psychosocial, Triglycerides, Self-report risk scale, MINI suicidal scale, Lipids

## Abstract

**Background:**

Biological factors have been associated with deliberate self-harm (DSH) but have not been integrated with clinical factors in routine risk assessments.

This study aimed to examine the incremental validity of lipid levels and platelet serotonin when combined with psychosocial factors in risk assessments for repeated admissions due to DSH.

**Methods:**

In this prospective observational study of 196 acutely admitted patients, results of blood tests performed upon admission and the MINI Suicidal Scale and psychosocial DSH risk factor assessments performed at discharge were compared with the incidence of DSH recorded during the first 3 and 12 months after discharge.

**Results:**

High triglyceride levels were found to be a significant marker for patients admitted 3 or more times due to DSH (repeated DSH, DSH-R) when tested against other significant risk factors. When all (9) significant univariate factors associated with 12-month post-discharge DSH-R were analyzed in a multivariate logistic regression, the MINI Suicidal Scale (p = 0.043), a lack of insight (p = 0.040), and triglyceride level (p = 0.020) remained significant. The estimated 12-month area under the curve of the receiver operator characteristic (ROC-AUC) for DSH-R was 0.74 for triglycerides, 0.81 for the MINI, 0.89 for the MINI + psychosocial factors, and 0.91 for the MINI + psychosocial factors + triglycerides. The applied multifaceted approach also significantly discriminated between 12-month post-discharge DSH-R patients and other DSH patients, and a lack of insight (p = 0.047) and triglycerides (p = 0.046) remained significant for DSH-R patients in a multivariate analysis in which other DSH patients served as the reference group (rather than non-DSH patients).

**Conclusion:**

The triglyceride values provided incremental validity to the MINI Suicidal Scale and psychosocial risk factors in the assessment of the risk of repeated DSH. Therefore, a bio-psychosocial approach appears promising, but further research is necessary to refine and validate this method.

## Background

Many risk factors contribute to suicide and self-harming behavior [1]. Performing a full risk assessment of such behavior is therefore time-consuming and requires expertise [2]. However, acute settings are characterized by time pressure and high patient turnover. Screening instruments, such as the MINI Suicidal Scale, have been shown to help identify patients at risk for self-harm [3], and a 27-item scale based on 154 items from a collection of suicidal assessment instruments showed promise for the development of a new scale evaluating suicidal risk in settings in which time is limited [4].

Patients who are repeatedly hospitalized because of deliberate self-harm represent a special challenge for emergency units [5]. In a study of patients admitted to emergency wards after a suicide attempt, the Suicide Assessment Scale (SUAS, a 20-item scale) was found to perform well in screening for repeated suicide attempts in patients receiving ongoing psychiatric treatment but less well in screening for repeated suicide attempts in the entire study population [6]. A recent multicenter study showed that clinical decision rules based on 5 variables (gender, current psychiatric treatment, previous self-harm, antidepressant treatment, and/or self-poisoning with benzodiazepines) could be used to enhance risk assessment of repeated deliberate self-harm (DSH-R) in patients admitted to emergency units [7].

Biological factors have been associated with suicidal behavior [8,9], and it has been suggested that a model that integrates feasible biological markers and clinical risk factors could optimize the assessment of suicide risk [10]. Meta-analyses have shown that the 5-hydroxyindolacetate levels in cerebrospinal fluid and a positive dexamethasone test (DST) associated with mood disorders are significant markers of prospective suicide risk and that combining these two methods increases predictive accuracy [10]. One study demonstrated that serum cholesterol concentrations may be combined with DST results to provide a clinically useful estimate of suicide risk in depressed patients [11]. However, with the exception of total cholesterol, these tests are difficult to integrate into routine clinical practice in acute settings. Many cross-sectional and retrospective studies have revealed a significant correlation between lipids and self-harm [12-16]. However, two prospective studies found no associations between serum cholesterol levels in depressed patients and subsequent suicide attempts [17,18].

In a prospective study examining different screening methods for deliberate self-harm risk during the first year after discharge from an emergency psychiatric ward, we found that (i) high triglyceride (TG) levels [19], (ii) the MINI Suicidal Scale [3], and (iii) patients’ own estimates of future risk (Self-report Risk Scale) [20] were significant predictors of self-harming behavior following discharge from an emergency psychiatric ward. The present research aimed to test the incremental validity of a biological marker (TG levels) when combined with other risk factors to identify patients at risk for repeatedly being hospitalized due to self-harming behavior.

## Methods

An observational follow-up study was performed at the acute psychiatric ward of Aalesund Hospital in Norway, which serves a population of 125,000 individuals. The target group consisted of all patients who were acutely admitted from March 7, 2006, to March 7, 2007. Of these 489 patients, 254 (52%) provided written consent and were included in the study. Fifty-eight patients were lost to follow-up after discharge. Thus, the final study group consisted of 196 patients (40%) who were followed-up 1 year after discharge. The project was approved by the Regional Committee for Medical Research Ethics and the Ministry of Health and Care. Further information concerning the study methodology is included in previous reports [3,19].

Consenting patients provided a fasting blood sample 1 to 3 days after admission. At discharge, the patients’ therapists in the acute ward scored them based on the MINI Suicidal Scale and the Self-report Risk Scale. Other baseline variables were obtained from medical records.

Follow-up at 3 and 12 months after discharge was conducted by the patients’ therapists at outpatient psychiatric clinics or district psychiatric wards. Patients discharged to the community were monitored by their psychiatric nurse in the acute ward. Self-harm episodes were recorded during the 0–3-, 4–6-, 7–9-, and 10–12-month periods. Information was collected from patients and collaterals and from inpatient and outpatient hospital records.

### Baseline variables

Lipids and serotonin: Total cholesterol, low-density lipoprotein (LDL), high-density lipoprotein (HDL), and TG levels were measured in fasting blood serum, and serotonin was measured in blood platelets [19]. The fasting period was from 12:00 PM until blood was drawn at 8:00-9:00 AM the next morning.

The MINI Suicidal Scale is a subscale of the MINI neuropsychiatric interview [21]. We used a short, 6-item version of this instrument. Each item is scored yes = 1 or no = 0. Items 1-5 evaluate whether an event has occurred during the last month. The 6 included items are as follows: (1) do you think that you would be better off dead or wish you were dead; (2) do you want to harm yourself or to hurt or injure yourself; (3) do you think about suicide; (4) do you have a suicide plan; (5) have you attempted suicide; and (6) in your lifetime, did you ever make a suicide attempt? The number of “yes” responses to the MINI items (0-6) was recorded for further analyses.

Self-report Risk Scale (SRS): Because of the lack of available instruments, the SRS was constructed for this study to measure patients’ judgment regarding their own subsequent risk of self-harming behavior [20]. Two questions were addressed to the patients at discharge: what is your opinion of the risk that you (A) will try to hurt or injure yourself without the intention of killing yourself, and (B) will try to kill yourself? For each question, the patient was given 7 response options: no risk, low risk, moderate risk, high risk, very high risk, do not know the risk, or will not answer about the risk. In this study, no risk was scored as 0 (negative), and the other 6 response options were scored as 1 (positive).

Information regarding gender, age, length of hospital stay, legal status, and ICD-10 diagnosis was obtained from hospital records. Violent behavior and violent victimization were recorded during follow-up in a parallel project performed at the same time [22] and determined from the hospital records, as were some items from the Violence Risk-10 (V-RISK-10) screening tool, including V6, a lack of insight into their illness; V9, unrealistic planning for the future; and V10, future stress exposure/vulnerability. Hopelessness has been identified as an important risk factor for self-harm [23], and at discharge, the patients’ experience of hopelessness for their own future was scored as “yes” or “no” by the therapist.

### Outcome variables

The outcome measures included in the analysis were threats and attempts of suicide and threats and acts of non-suicidal self-injury (NSSI). A suicide attempt was defined as self-infliction of physical injury with the intention of killing oneself, and NSSI was defined as inflicting physical injury upon oneself without the intention of killing oneself [24]. Suicide attempts and NSSI were categorized as less severe (without the need for hospitalization) or severe (followed by hospitalization or fatality). All study subjects were scored as “No episodes”, “Yes – episode(s)”, or “Do not know”. To improve the statistical power, the analyses were performed by merging threats, less-severe acts, and severe acts of suicide and NSSI into one common variable: deliberate self-harm (DSH), which was coded as 0 (no self-harm), or 1 (suicidal or NSSI threat or attempt).

Some of the patients had both suicidal and NSSI behaviors at different times during follow-up, and it was difficult to categorize these behaviors into either the suicidal or NSSI group. Consequently, the patients were categorized into 3 groups; suicidal group, NSSI group, and suicidal + NSSI group [3,25].

Results from the 4–6-month and 7–9-month follow-up periods did not add significant information. For clarity, results are only provided for short-term (0–3 months) and long-term (0–12 months) periods. Because psychosocial and biological risk factors may change over time, both the short- and long-term periods are of interest.

### Statistical analysis

The data were analyzed using SPSS version 19.0. Chi-square tests were used to compare frequencies, and t-tests and one-way analysis of variance (ANOVA) were used for continuous variables. Binary logistic regression analyses were conducted to obtain univariate and multivariate effect sizes (odds ratio, OR). A block-enter procedure was used to monitor the progression of the chi-square test to examine whether each of the factors significantly added to the variance explained by the model. To address the relative importance of the various contributing factors, two R^2^ approximations (Cox & Snell R^2^ and Nagelkerke R^2^) were employed as the lower and upper limits of the extent to which total variance in DSH could be explained by the predictors.

Analysis of the area under the curve (AUC) of the receiver operating characteristic (ROC) was performed to assess the overall predictive accuracy of the models. The following predictive validity estimates were also computed: sensitivity (how many of the DSH patients displayed a positive test), specificity (how many patients without DSH displayed a negative test), positive predictive value (PPV, how many patients with a positive test exhibited DSH), negative predictive value (NPV, how many patients with a negative test did not exhibit DSH), number needed to assess (NNA, how many patients would need to be assessed to identify one true DSH patient, which is equal to 1/PPV), and the likelihood ratio (LR). The LR determines the extent to which the odds of an outcome (e.g., DSH) increase when a test is positive (LR+) and decrease when a test is negative (LR–). An LR + value of 3.0 signifies a 3-fold increase in the likelihood of DSH. For tests with only two outcomes (DSH or not), the LR + and LR– can be expressed as sensitivity/(1 – specificity) and (1-sensitivity)/specificity, respectively [26].

## Results

A comparison between the follow-up sample (40%) and the other admitted patients (60%) is shown in Table 1.

**Table 1:** Comparison of follow-up and missing patients

	**Follow-up (*n *= 196)**	**Missing (*n *= 293)**	**p-value**
Men	104 (53%)	158 (54%)	0.820
Mean age (years)	42.4	45.7	0.027
Mean hospital stay (days)	20	14	<0.001
Involuntary admittance	42 (21%)	63 (23%)	0.655
Mandatory aftercare	17 (8.6%)	22 (7.5%)	0.799
F1x substance abuse	30 (15%)	44 (15%)	0.898
F10 alcohol abuse	21 (11%)	29 (10%)	0.780
F2x psychotic disorders	29 (15%)	49 (17%)	0.703
F30-31 bipolar disorders	29 (15%)	24 (8.2%)	0.049
F32-39 depressive disorders	54 (28%)	66 (23%)	0.213
F4x anxiety disorders	38 (19%)	55 (19%)	0.704
F6x personality disorders	13 (6.6%)	13 (4.5%)	0.319
Feeling of hopelessness	24 (12%)	33 (14%)	0.484
DSH during hospital stay	14 (7.1%)	0	<0.001
Positive SRS rating	35 (20%)	28 (12%)	0.043
Positive MINI items at discharge (0-6)	2.4	1.8	0.004
Suicide attempt prior to admission	29 (15%)	24 (10%)	0.076
Suicide attempt ever	84 (45%)	67 (28%)	<0.001
Suicidal ideation prior to admission	92 (49%)	102 (42%)	0.130
Wish of self-harm prior to admission	73 (39%)	71 (29%)	0.031
Total cholesterol (mmol/liter)	5.0	5.1	0.876
Triglycerides (mmol/liter)	1.4	1.3	0.527
HDL (mmol/liter)	1.3	1.4	0.083
LDL (mmol/liter)	3.3	3.6	0.227
Platelet serotonin^a^	3.5	3.2	0.685
Platelet serotonin^b^	0.85	0.82	0.922

DSH = deliberate self-harm; SRS = Self-report Risk Scale; MINI = the MINI Suicidal Scale; HDL = high-density lipoprotein; LDL = low-density lipoprotein.

^a^nmol/10^9^ platelets, not using serotonin reuptake inhibitors (SSRI, SNRI or TCA).

^b^nmol/10^9^ platelets, using serotonin reuptake inhibitors.

Two patients took their lives during the follow-up [3], and 75 (36%) patients were recorded as exhibiting DSH at the 1-year follow-up, 52 of whom were hospitalized again because of DSH. Among these patients, 23 were re-hospitalized once; 11 underwent 2 re-hospitalizations; 7 experienced 3 re-hospitalizations; 5 underwent 4 re-hospitalizations; and 6 patients were subjected to 5 re-hospitalizations related to DSH. Patients exhibiting 3 or more DSH-related re-hospitalizations (repeated DSH, DSH-R) constituted 24% of the total sample of DSH patients and 9% of all patients but accounted for 83% of DSH-related re-hospitalizations and 44% of all re-hospitalizations. One DSH-R patient was recorded as only exhibiting suicidal attempts. The other 17 DSH-R patients were recorded as displaying at least 1 suicidal attempt and 1 NSSI at different times (suicidal + NSSI group). A comparison of the non-DSH patients, DSH-R patients, and patients with 1 or 2 DSH re-hospitalizations or post-discharge DSH episodes without hospitalization (1-2 DSH) is presented in Table 2. DSH-R was recorded for 13 patients within the first 3 months after discharge.

**Table 2:** Comparison of patients not showing deliberate self-harm (DSH) with patients exhibiting one or two DSH episodes and those displaying repeated DSH after discharge

**Number of patients (*N *= 196)**	**No DSH**	**1-2 DSH**	**DSH-R**
	***n *= 121 (62%)**	***n *= 57 (29%)**	***n *= 18 (9%)**
Males/females (%)	59/60 (50/50%)	36/24 (60/40%)	6/12 (33/67%)^b*^
Mean age, years [95% CI]	43 [40-46]	42 [38-45]	38 [31-45]
Mean hospital stay, days [95% CI]	22 [ 8-27]	20 [16-24]	12 [6-18]^a*^
Involuntary admissions	25 (21%)	12 (20%)	5 (28%)
Mandatory aftercare	10 (8.4%)	6 (10%)	1 (5.6%)
Inpatient DSH	4 (3%)	3 (5%)	7 (39%)^a***b***^
Hopelessness at discharge	11 (10%)	7 (13%)	6 (38%)^a** b*^
Lack of insight“	41 (35%)	30 (52%)^a*^	15 (83%)^a*** b*^
Unrealistic plans“	43 (37%)	35 (69%)^a**^	13 (72%)^a**^
Stress exposure“	61 (53%)	37 (64%)	11 (61%)
Positive MINI items [95%CI]“	1.9 [1.5-2.3]	3.0 [2.3-3.6]^a**^	4.1 [3.3-4.9]^a***b*^
Positive SRS ratings“	33 (30%)	26 (50%)^a*^	15 (83%)^a*** b*^
Violent offenders after discharge	21 (18%)	15 (25%)	8 (44%)^a*^
Victims of violence“	19 (17%)	14 (24%)	10 (56%)^a*** b*^
**Main diagnoses**			
Substance misuse (F10-19)	19 (16%)	8 (13%)	3 (17%)
Psychoses (F20-29)	18 (15%)	9 (15%)	2 (11%)
Manic/bipolar disorders (F30-31)	19 (16%)	8 (14%)	2 (11%)
Depressive disorders (F32, F34.1)	37 (26%)	26 (15%)	0 ^a** b* f^
Anxiety disorders (F40-49)	24 (20%)	11 (18%)	3 (17%)
Personality disorders (F60-62)	3 (3%)	3 (5%)	7 (39%)^a*** b*** c^
**Blood measures**			
Total cholesterolmmol/l [95%CI]	4.9 [4.7-5.1]	5.3 [4.9-5.6]	5.3 [4.8-5.9]
LDL“	3.2 [3.0-3.3]	3.6 [3.3-3.9]	3.6 [3.0-4.1]
HDL“	1.3 [1.2-1.4]	1.3 [1.1-1.5]	1.2 [1.0-1.4]
Triglycerides“	1.2 [1.1-1.3]	1.4 [1.2-1.6]	2.1 [1.5-2.6]^a***b*^
Platelet serotonin^d^	3.3 [2.6-3.8]	3.7 [2.4-5.1]	3.0 [1.9-4.1]
Platelet serotonin^e^	0.88 [0.53-1.2]	0.72 [0.38-1.1]	0.79 [0.27-1.6]

DSH = deliberate self-harm; 1-2 DSH = one or two DSH episodes/re-admissions; R-DSH = three or more DSH re-admissions.

*p ≤ 0.05, ** p ≤ 0.01, ***p ≤ 0.001.

^a^Significantly different from non-DSH patients.

^b^Significantly different from 1-2 DSH patients.

^c^All unstable (borderline) personality disorders.

^d^nmol/10^9^ platelets [95% CI], not using serotonin reuptake inhibitors (SSRI, SNRI, or TCA).

^e^nmol/10^9^ platelets [95% CI], using serotonin reuptake inhibitors.

^f^Two of the DSH-R patients had co-morbid diagnosis of unipolar depression.

For patients with suicidal behavior only, the odds ratios [95% CIs] for total cholesterol and TG associated with 12-month suicidal behavior were 1.3 [0.95-1.8] (p = 0.104) and 1.9 [1.1-3.5] (p = 0.026), respectively. The corresponding values for 12-month NSSI only were 1.2 [0.66-2.3] (p = 0.524) and 1.8 [0.69-4.7] (p = 0.234); for 12-month suicidal behavior + NSSI, the odds ratios for total cholesterol and TG were 1.3 [0.89-1.9] (p = 0.171) and 2.6 [1.4-4.9] (p = 0.003), respectively.

Eleven of the 18 DSH-R patients had 1 or 2 co-morbid psychiatric diagnoses, such as alcohol disorders (3 patients), mild intellectual disability (3), personality disorders (1 borderline and 1 unspecified), bipolar depression (2), unipolar depression (2), and anxiety disorders (2).

### Univariate and multivariate predictive values

TG levels significantly predicted DSH-R at 12 months, with an OR [95% CI] of 4.2 [1.9-9.4] (p < 0.001). The 3-month OR was 3.2 [1.7-6.3] (p = 0.001). No other lipids were significant in the 1-2 DSH and DSH-R groups, and the platelet serotonin levels were also not significant.

The 3- and 12-month ORs [95% CIs] for the MINI for DSH-R were 1.7 [1.3-2.4] (p = 0.001) and 1.8 [1.3-2.4] (p < 0.001), respectively, and the corresponding values for SRS were 12 [2.5-54] (p = 0.002) and 12 [3.2-44] (p < 0.001).

When the 9 significant factors associated with post-discharge DSH-R at 12 months (see Table 2), including gender, hopelessness, inpatient DSH, a personality disorder (PD), violent victimization, a lack of insight, positive MINI items, SRS, and TG, were entered into multiple regression analyses, the lack of insight and TG were the only factors that remained significant.

Table 3 shows the results of the block-entry logistic regression analysis of the 9 significant factors and age. Age was not significant in the univariate analysis but is considered to be significantly associated with self-harm; therefore, age was entered into the analysis as the tenth factor. Gender, age, PD, and TG were entered in the first step of the analyses; hopelessness, inpatient DSH, victims of violence, and lack of insight were added in the second step; and MINI and SRS were added in the third step.

**Table 3:** Stepwise multivariate analyses of the significant factors of patients with three or more re-hospitalizations caused by DSH 12 months after discharge of index admission

			**Variance**		**Explained**
	**OR [95% CI]**	**p**	***χ***^***2 ***^***(d.f.)***	**p**	**Variance**^**a**^
**Step 1**					
Female	1.4 [0.32-6.5]	0.638			
Age	1.0 [0.96-1.05]	0.696			
Personality disorders	36 [4.8-274]	<0.001			
Triglycerides	3.4 [1.5-7.6]	0.004			
Total variance of step 1			33 (4)	<0.001	24-44%
**Step 2**					
Female	0.58 [0.03-12]	0.729			
Age	1.1 [0.97-1.2]	0.168			
Personality disorders	190 [1.1-3200]	0.045			
Triglycerides	7.8 [1.6-37]	0.010			
Lack of insight	29 [1.5-570]	0.025			
Victims of violence	19 [1.2-320]	0.036			
Hopelessness	12 [0.69-240]	0.087			
Inpatient DSH	63 [0.88-4600]	0.058			
Increase from step 1 (hopelessness, victims of violence,lack of insight, inpatient DSH)			29 (4)	<0.001	17-31%
Total variance of step 2			62 (8)	<0.001	41-75%
**Step 3**					
Female	13 [0.59-280]	0.353			
Age	1.2 [0.97-1.5]	0.089			
Personality disorders	1100 [0.36-34x10^6^]	0.087			
Triglycerides	102 [0.76-14000]	0.064			
Victims of violence	2500 [0.52-12x10^6^]	0.070			
Lack of insight	2600 [0.52-13x10^6^]	0.071			
Hopelessness	1.4 [0.02-98]	0.872			
Inpatient DSH	1.1 [0.003-360]	0.978			
Self-report risk scale	0.51 [0.02-15]	0.699			
MINI suicidal scale	4.7 [0.99-22]	0.051			
Increase from step 2 (by MINI and self-report risk scale)			12 (2)	0.003	6-10%
Total variance of step 3			74 (10)	<0.001	47-85%

DSH, deliberate self-harm.

^a^“pseudo R^2^” = Cox & Snell R^2^ – Nagelkerke R^2^ estimates (of the factors’ contribution to the total variance).

In the 12-month block-entry regression with the same 10 factors and 1-2 DSH as the inclusion criteria for the reference group (versus non-DSH patients in the previous analysis), PD (OR [95% CI] = 9.2 [1.9-44], p = 0.006) and TG (2.5 [1.1-5.5], p = 0.022) were the only significant variables in Step 1. Inpatient DSH (29 [1.0-820], p = 0.047) and TG (3.1 [1.0-9.3], p = 0.049) were significant in Step 2, and lack of insight (28 [1.0-760], p = 0.047) and TG (3.7 [1.0-13], p = 0.046) were significant in Step 3.

Similar results were obtained in the block-entry regression analysis at 3 months post-discharge of DSH patients. High TG level was the only significant factor in the third step of the stepwise regression when non-DSH patients were used as the reference group, with an OR [95% CI] of 7.5 [1.0-55] (p = 0.048). No factors were significant in the 3. step of the multiple regression analysis when 1-2 DSH patients (within 3 months after discharge) were the reference group. The TG value in the third step was 3.5 [0.67-18] (p = 0.156).

No significant correlations were found between TG and the main diagnostic groups. Furthermore, the results did not change when controlling for diagnoses of diabetes or alcohol misuse and for current medication at the time of admission. Only 1 of the DSH patients was recorded as having a diagnosis of heart disease.

### Other predictive values

Finally, to further test possible incremental validity, TG levels were entered into the analysis together with positive MINI items (coded 0-6), female gender (coded 0, 1), PD (coded 0, 1), a lack of insight (coded 0, 1), violent victimization (coded 0, 1), and SRS (coded 0, 1). TG levels were coded as follows: 0-25th percentile = 2, 26th-50th percentile = 1, and 51st-100th percentile = 0. The ROC-AUC values associated with the 1-year follow-up for the 1-2 DSH and DSH-R groups are displayed in Table 4.

**Table 4:** The area under the curve of the receiver operator characteristic (AUC) for one-year DSH-R

	**Compared with non-DSH**		**Compared with 1-2-DSH**	
	**AUC [95% CI]**	**p**	**AUC [95% CI]**	**p**
TG	0.74 [0.61-0.86]	0.001	0.69 [0.55-0.83]	0.017
SRS	0.77 [0.65-0.88]	<0.001	0.68 [0.54-0.81]	0.027
MINI Suicidal Scale	0.81 [0.72-0.91]	<0.001	0.62 [0.48-0.75]	0.146
Other variables (OV)^a^	0.86 [0.74-0.97)]	<0.001	0.81 [0.69-0.93)]	<0.001
MINI + TG	0.85 [0.77-0.94]	<0.001	0.71 [0.57-0.85]	0.009
MINI + OV	0.89 [0.80-0.98]	<0.001	0.76 [0.64-0.89]	0.001
OV + TG	0.89 [0.82-0.97]	<0.001	0.87 [0.77-0.96]	<0.001
MINI + OV + SRS + TG	0.91 [0.83-0.99]	<0.001	0.82 [0.70-0.94]	<0.001
OV + SRS + TG	0.92 [0.85-0.98]	<0.001	0.88 [0.79-0.97]	<0.001

DSH = deliberate self-harm; DSH-R = repeated DSH; SRS = Self-report Risk Scale; TG = triglycerides;

^a^Female, personality disorder, victims of violence, lack of insight.

Other predictive values are displayed in Table 5. The cut-off values shown in Table 5 were based on the coordinates of all of the recorded sensitivities/specificities. For both models, the cut-off values constituted the largest sum of the sensitivity + specificity.

**Table 5:** **Comparison of predictive values and likelihood ratios for the MINI and the MINI + OV**^**a **^**+ SRS + TG for DSH-R patients**

	**Cut off**	**Sens**	**Spec**	**PPV**	**NPV**	**NNA**	**LR+**	**LR-**
MINI (0-6)	3.5	0.78	0.75	0.30	0.96	3.3	2.9	0.24
MINI + OV^a^ + SRS + TG (0-14)	8.5	0.78	0.92	0.64	0.96	1.6	9.8	0.24

MINI = the MINI Suicidal Scale; OV = other significant variables; SRS = Self-report Scale; TG = triglycerides; Sens = sensitivity; Spec = specificity; PPV = positive predictive value; NPV = negative predictive value; NNA = number needed to assess; LR^+^ = positive likelihood ratio; LR- = negative likelihood ratio.

^a^Female, personality disorders, victims of violence, lack of insight.

## Discussion

The main finding of the present study was that TG showed significant incremental validity in the risk assessment of DSH-R at 3 and 12 months after discharge. The applied bio-psychosocial approach, including the MINI Suicidal Scale, psychosocial risk factors, and TG, produced a PPV that was twice as high as that obtained using the MINI alone. Furthermore, TG enhanced the predictive validity of DSH when comparing DSH-R patients with 1-2 DSH patients. One possible explanation for this finding is that an elevated TG level reflects stress activation in the noradrenergic nervous system. Studies have shown that psychological distress can lead to increased TG levels [27-29]. The increase in TG following psychological distress results from direct psychological sympathetic activation and is not a metabolic effect [30]. Elevated TG levels have also been associated with general anxiety disorders [31]. Albrink et al. found a significant positive association between TG and obesity [32]. Stress has been identified as a common factor in obese individuals, and the neurobiology of stress overlaps significantly with that of appetite and energy regulation [33]. However, measurements of lipid levels after admission may not accurately reflect the lipid levels occurring prior to self-harm actions following discharge. Thus, monitoring TG levels near the time of stress exposure may improve the detection of this association.

Although there were no apparent differences in the risk of stress exposure between the 3 groups examined in this study at discharge, the DSH-R patients may have had a lower stress tolerance or less efficient coping skills. A lack of insight may contribute to increased vulnerability and a reduced ability to avoid stressful situations, which may also be the case for individuals with unstable personality disorders [34]. Furthermore, stress or stress-related conditions or symptoms or the risk of exposure to future stressful situations may have been ignored by hospital staff when rating the “risk of stress exposure” (V10) at discharge. Identifying a subgroup with a high risk of self-harm resulting from increased psychological distress may be of clinical importance both for treatment (coping with and reducing internal stress) and prevention (reducing external environmental stress). Regarding prevention, during the 1-year follow-up, victimization was increased in the DSH-R group compared with the other subgroups, indicating increased stress exposure, stress reactions, and stress vulnerability, which is in agreement with earlier reports of an association between victimization and DSH [35,36].

Although depression and psychosis are strong risk factors for self-harm [1], these factors were significantly underrepresented in the DSH-R group and in all self-harming patients in this study [3]. However, several factors may explain these results: (i) there was a high prevalence of self-harm in this study; (ii) patients with depression or psychoses are known to be at self-harm risk, and these patients were not discharged before the risk was considered low and these patients may have received sufficient treatment and self-harm monitoring after being discharged; and (iii) patients with alcohol abuse or personality disorders and repeated admissions may have been treated inadequately, or co-morbidity may have been overlooked, in which case self-harm risk may have been ignored [37].

Previous studies revealed a negative correlation between body mass index (BMI) and suicide [38,39]. However, one recent investigation conducted in Taiwan demonstrated increased suicide risk in both underweight and obese individuals [40]. Thus, the interpretation and understanding of this association are still inadequate. A greater body weight reduces the fatality of poisoning and shifts suicidal acts away from lethal means such as hanging, but it remains difficult to exclude other possible explanations for the reported correlation, such as psychiatric disorders that could lead to weight loss and suicidal ideation [41], including depression or anorexia. Our finding of an association between high TG and DSH-R is contrary to the BMI-related “protective” effect but concurs with results of studies that have found a potentially positive association between obesity and stress [33].

Other research has addressed the importance of focusing on high sensitivity in screening for self-harm repeaters in emergency settings [7]. The direct clinical application of AUC and OR values can be difficult. Other predictive values may add important information regarding the clinical applicability of screening tools. For most risk assessment instruments, the identification of individuals at risk is acceptable (high sensitivity), but the total predictive value is often low, with many false positives (low specificity). Compared with the MINI Suicidal Scale, the applied multifaceted bio-psychosocial screening method exhibited increased specificity, as demonstrated by a decrease in NNA values from 3.3 to 1.6. This finding indicates an increased positive predictive value and implies that a total of 2.3 or 0.6 patients, respectively, would have been unnecessarily assessed for DSH-R risk for every true DSH-R patient identified. The increase in the positive likelihood ratio from 2.9 to 9.8 indicates that a positive MINI test result increases the likelihood of a patient exhibiting DSH-R by 2.9-fold, whereas the increase in likelihood is 9.8-fold for a positive bio-psychosocial test. Our results suggest that easily accessible bio-psychosocial risk factors could be employed to screen for DSH risk in emergency settings.

### Limitations

Only one hospital was involved in the present work, and the study sample was small, consisting of 40% of the potential subjects. There were some differences between the study sample and the other patients (non-consenting patients and patients lost to follow-up). Moreover, our sample of DSH-R patients was small, and the study population was selective relative to the community cohorts included in various BMI studies [38,39]. These factors limit the generalizability of the obtained results. However, there was a higher incidence of self-harm-related variables in the follow-up sample. Records of self-harm behavior can be difficult to obtain and may suffer from under-reporting. Although the raters (baseline variables) and recorders (outcome variables) involved in the present study were responsible for different parts of the research, they all worked in the same clinical department. While the recorders were blinded to the baseline measures and ratings, there may still have been some communication that could have threatened the ideal performance of independent measurements. However, the clinicians’ familiarity with the patients may have counteracted the underreporting of self-harm episodes. Somatic illnesses that may influence lipid levels, such as heart disease and diabetes, could have been underreported in the psychiatric records, even though the patients were treated during their stay in the ward.

Alcohol consumption, cigarette smoking, and obesity increase TG levels [32,42-44]. Beyond recording the ICD-10 diagnosis of alcohol abuse, these factors were not controlled for in the study. However, smoking decreases BMI and counteracts the alcohol-related increase in TG levels (Wannamethee & Shaper, 1992), and there is no available evidence of interaction effects between alcohol use, smoking, and BMI in relation to suicide risk [40]. We did not control for dietary patterns or stress-related dietary changes, such as high carbohydrate intake, that may have caused elevated TG levels [33,45], and no anxiety scales or tools were used in the study beyond the diagnosis of anxiety disorders.

## Conclusions

In summary, TG levels explained variability in self-harming behavior that was not explained by the other risk factors assessed. This finding may help to identify a subgroup of patients characterized by repeated self-harming behavior. Thus, a bio-psychological approach appears to be promising, at least for high-risk subgroups. Further studies controlling for anxiety and stress-related measures, obesity measures, and alcohol consumption and smoking patterns are necessary to further develop and validate this model.

## Abbreviations

BMI: Body mass index; CI: Confidence interval; DSH: Deliberate self-harm; ICD-10: International classification of diseases – version 10; LR: Likelihood ratio; MINI: The MINI international neuropsychiatric interview; NSSI: Non-suicidal self-injury; OR: Odds ratio; PPV: Positive predictive value; ROC-AUC: Area under the curve of the receiver operator characterstic; SRS: Self-report Risk Scale; TG: Triglycerides.

## Competing interests

The authors declare that they have no competing interests.

## Authors’ contributions

JOR and SB contributed to the design of the study. JOR carried out the data analyses. JOR, OLM and SB contributed significantly to the interpretation of the data, to the preparation of the manuscript, and to the final approval of the manuscript. All authors read and approved the final manuscript.

## Pre-publication history

The pre-publication history for this paper can be accessed here:

http://www.biomedcentral.com/1471-244X/14/54/prepub
